# Comparison of Clinical and Radiological Results of Lateral Retinacular Release or Lateral Retinacular Lengthening Methods Combined With Medial Retinaculum Plication in Patellofemoral Instability

**DOI:** 10.7759/cureus.29684

**Published:** 2022-09-28

**Authors:** Murat Saylik, Yücel Bilgin, Teoman Atıcı

**Affiliations:** 1 Department of Orthopedics and Traumatology, Vocational School of Therapy and Strengthening, Mudanya University, Bursa, TUR; 2 Department of Orthopedics and Traumatology, Faculty of Medicine, Bursa Uludağ University, Bursa, TUR

**Keywords:** lateral lengthening, medial plication, lateral release, patellofemoral joint, instability

## Abstract

Introduction

In this study, we aimed to compare the clinical and radiological results of patients who underwent medial retinaculum plication (MRP) combined with lateral retinacular release (LRR) or lateral retinacular lengthening (LRL) with the diagnosis of patellofemoral (PF) instability.

Methods

In our study, we retrospectively analyzed 75 knees of 75 adult patients (43 females and 32 males) who underwent MRP+LRR or MRP+LRL due to PF instability without osseous pathologies. Patients were divided into two groups (MRP+LRR and MRP+LRL) according to the surgical method. The clinical and radiological results of the two groups were compared.

Results

MRP+LRL surgery was performed on 45 knees and MRP+LRR surgery on 30 knees. The mean age was 26.5 (18-43) years. There was no significant difference between the two groups in the change in patellar lateral shift (PLS) (p=0.429) and congruence angle (CA) (p=0.218) values. However, there was a significant difference between the two groups in the change in patellar tilt angle (PTA) (p=0.009) and lateral patellofemoral angle (LPFA) (p<0.001) values. The change in PTA and LPFA values was higher in the MRP+LRL group. There was no significant difference between the two groups in terms of pre-operative and post-operative Lysholm knee scoring scale (p=0.205, p=0.228), Kujala pain scale (p=0.393, p=0.596), and Tegner activity level scale values (p=0.121, p=0.899).

Conclusions

MRP+LRR or MRP+LRL provided successful results for correcting the instability in PF instability without osseous pathologies such as patella alta, tibial tubercle-trochlear groove (TT-TG) dysplasia, trochlea dysplasia, genu valgus, and tibial-femoral torsion. While PTA and LPFA values improved more with the MRP-LRL method, clinical results were similar in both methods.

## Introduction

The term patellofemoral (PF) instability refers to the abnormal position of the patella in any plane. PF stability depends on the alignment of the lower extremities; the anatomical compatibility of the articular surfaces of the patella and trochlea, which are static stabilizers; and the adequacy of the medial patellofemoral ligament (MPFL) and quadriceps muscle group, which are dynamic stabilizers [1]. The primary factors causing PF instability are trochlear dysplasia, quadriceps and vastus medialis oblique (VMO) dysplasia, patella alta, MPFL and medial retinacular ligament (MRL) insufficiency, tensor fascia lata contracture, and lateralization of the tuberositas tibia. Secondary factors are grouped as rotational deformity of the lower extremity, genu valgum, genu recurvatum, and syndromes that cause hereditary ligament laxity (Ehlers-Danlos syndrome, Marfan syndrome, etc.) [2, 3]. PF instability occurs with multiple anatomical incompatibilities; therefore, many different surgical treatment combinations have been reported [4]. These surgical interventions are grouped under proximal realignment surgery, distal realignment surgery, and proximal/distal realignment surgery. Proximal realignment surgery consists of interventional methods performed on the medial and lateral parts of the PF joint. Lateral retinacular release (LRR) or lateral retinacular lengthening (LRL) methods which are combined with medial retinacular plication (MRP), are included in the proximal realignment surgery group [5].

The lateral retinaculum is effective in the lateral balance of the patella. Surgeries for the lateral retinaculum (LRR/LRL) aim to regulate the lateral tensile strength balance and the extensor mechanism [6]. LRR was first described by Merchant and Mercer in 1974 and has been applied in isolation or in combination with surgical methods that provide medial stability of the patella until today [7]. LRL is also effective in the lateral patellofemoral angle (LPFA) and the centralization of the patella in the trochlear sulcus, regardless of the surgical method to be applied to the medial stability of the patella [8]. MRP can be applied in cases of acute patellar dislocations where conservative treatment fails, recurrent dislocations after low-energy trauma, recurrent patellar subluxation, mild patellar lateralization due to medial retinacular weakness, and patellar lateralization due to ligament laxity. It is contraindicated in cases of advanced trochlear dysplasia, Q angle greater than 20 degrees, patella alta, and congenital patellar dislocation [9].

Our study aimed to compare the radiologic and functional results of the cases where the adolescent development process was completed. We applied MRP+LRR or MRP+LRL due to PF instability and investigated the effect of patella and trochlea morphology on these results.

## Materials and methods

Our study retrospectively evaluated 112 knees of 112 patients who underwent MRP+LRR or MRP+LRL due to PF instability between April 2015 and February 2020 and whose epiphyseal line was closed. Approval for the study was obtained from the local ethics committee with protocol number 367, dated February 22, 2021. Informed consent was obtained from all patients. Inclusion criteria for the study were patients diagnosed with chronic patellar subluxation, with at least three episodes of subluxation, positive J sign, unsuccessful conservative treatment for more than 12 months, and minimum one-year postoperative follow-up. Exclusion criteria included patients with patella alta (Caton-Deschamps index >1.3) with lower extremity rotational deformity, hereditary connective tissue laxity (Ehlers-Danlos syndrome, Marfan syndrome, etc.), genu valgus, tibial tuberosity-trochlear grove (TT-TG) distance of more than 20 mm, and those patients who had undergone previous surgery for the knee joint and who developed PF instability after severe knee trauma.

**Figure 1:**
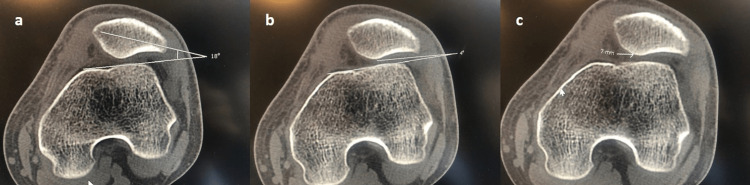
a) Lateral patellofemoral angle (LPFA) radiological measurement of the patient, b) Patellar tilt angle (PTA) radiological measurement of the patient, c) Patella lateral shift (PLS) radiological measurement of the patient.

Seventy-five knees (43 females and 32 males) of 75 patients who could be followed up postoperatively until a minimum of 12 months were included in the study. Patients were divided into two groups (MRP+LRR and MRP+LRL) according to the surgical method. MRP+LRL surgery was performed on 45 knees and MRP+LRR surgery on 30 knees. Patients were informed about both treatment methods preoperatively. The patient and the orthopedic specialist decided together which method would be applied. Then, the clinical and radiological results of the two groups were compared. In clinical evaluation, pre-operative and post-operative first year "Kujala pain scale," "Tegner activity level," and "Lysholm knee scale" values were used. In the radiological evaluation, standard anteroposterior (AP) and lateral radiographs, merchant radiographs of the knee at 30 degrees of flexion, and CT and MRI of the knee at 15 degrees of flexion were used preoperatively and in the first year postoperatively. Patellar tilt angle (PTA) (Figure 1a), LPFA (Figure 1b), patella lateral shift (PLS) (Figure 1c), and congruence angle (CA) were measured using same level MR (had to be evaluated for the presence of chondral damage before surgery) or CT axial sections passing through the midline of the patella. Using the Wiberg classification for patella morphology, the effect of three types of patella morphology on functional and radiological outcomes in the treatment of PF instability was investigated [10]. Dejour D et al.'s classification is widely used to identify trochlea morphology [11]. In our study, the effect of trochlea morphologies determined according to Dejour classification on functional and radiological results in treating PF instability was investigated.

The knees that we performed revision surgery on were accepted as the recurrences of PF instability. A single surgeon performed all surgical procedures.

Surgical technique

First, knee arthroscopy was performed from the anterolateral and anteromedial portals and, if necessary, from the superolateral and superomedial portals for the diagnosis and treatment of intra-articular pathologies. The development of PF instability with knee flexion extension, chondral damage in the PF joint (Outerbridge classification was used), and menisci and cruciate ligaments were evaluated with arthroscopy. MRP+LRL and MRP+LRR surgeries were performed if there was no chondral damage in the PF joint or the damage was stage 1-2 according to the Outerbridge classification.

In LRL, the lateral retinaculum was reached by applying a parapatellar longitudinal 5-6 cm incision. A vertical incision was made approximately 1 cm lateral to the patella to the superficial longitudinal fibers of the lateral retinaculum. Superficial longitudinal fibers and deep, transverse fibers were separated from this incision site. Next, a vertical incision was made to the deep, transverse fibers anterior to the femoral epicondyle attachment site. The posterior of the superficial fibers and the anterior of the deep fibers were combined with a non-absorbable suture (No: 2 Ethibond, Ethicon Inc, Somerville, NJ) to lengthen the lateral retinaculum between 1 and 2 cm. In LRR, the vastus lateralis was preserved with a hook-shaped radiofrequency probe under the guidance of arthroscopy, and the lateral retinaculum between the vastus lateralis obliquus and the lateral femoral epicondyle was released full-thickness. Using the radiofrequency probe was an attempt to prevent hematoma formation, the most common complication in the literature.

Surgical treatment of MRP began with a medial parapatellar longitudinal 4 cm incision. The VMO and medial retinaculum were separated along the medial border of the patella and transferred laterally with a non-absorbable suture (No: 2 Ethibond, Ethicon Inc, Somerville, NJ).

The tourniquet was opened, and the centralization of the patella in the trochlear groove was checked with suture tension and arthroscopic imaging. Active PF joint adjustment and ligament balance were checked by flexing the knee at 90 degrees several times.

Post-surgical follow-up protocol

In both patient groups, mobilization was started in all patients with a hinged knee brace (at an angle to allow 60 degrees of flexion and 0 extensions) and a single cane on the first postoperative day and with isometric quadriceps exercises. On the 15th day, the flexion angle was increased to 90 degrees. Light resistance exercises were started, and standing with a single cane was continued. In the first month, the patients began the rehabilitation program with 120 degrees of flexion, standing unsupported, and using a simple knee compression. Patients were allowed to return to normal daily life after two months and to active sports after six months.

Statistical analysis

Frequency, mean, and SD values were calculated by descriptive statistical methods. A T-test was used to evaluate the differences between the two groups. ANOVA test was used to evaluate the effect of patella type on the results. The analyses considered a two-tailed hypothesis, and differences were accepted as significant if the p-value was 0.05 or less. SPSS 18.0 software for Windows (SPSS, Inc., Chicago, IL, USA) was used to evaluate statistical analyses.

## Results

In our study group, 57.33% (n=43) were female and 42.67% (n=32) were male. Thirty knees (18 female, 12 male) treated with MRP+LRR and 45 knees (25 female, 20 male) treated with MRP+LRL were compared. The mean age was 26.5 years (range: 18-43). The mean age in the MRP+LRR group was 32.24 (range: 18-43), and the mean age in the MRP+LRL group was 26.88 (range: 19-38) (p=0.343). The mean follow-up period was 40.65 months (range: 14-70). The mean follow-up time was 41.35 months (range: 16-70) in the MRP+LRR group and 39.80 months (range: 14-68) in the MRP+LRL group (p=0.807).

Radiological evaluation

There was a significant difference between the two groups in terms of PTA change before and after surgery (p=0.009) (Table 1). There was no significant difference between the two groups in terms of PLS change before and after surgery (p=0.429) (Table 1). There was a significant difference between the two groups in terms of LPFA change before and after surgery (p<0.001) (Table 1). In the MRP+LRR group, the mean pre-operative CA was 25.83 (range: 15-42), and the post-operative CA was 3.83 on average (range: -2-8). There was a significant decrease observed (p=0.145). In the MRP+LRL group, the mean pre-operative CA was 24.6 (range: 17-34), and the post-operative CA was 3.51 on average (range: -3-10). A significant decrease was observed (p=0.218). There was no significant difference between the two groups in terms of CA change before and after surgery (p=0.348).

**Table 1:** Comparison of patellar tilt angle, patella lateral shift, and lateral patellofemoral angle changes according to groups. PTA: Patellar tilt angle; PLS: Patella lateral shift; LPFA: Lateral patellofemoral angle; MRP+LRL: Medial retinaculum plication and lateral retinacular lengthening; MRP+LRR: Medial retinaculum plication and lateral retinacular release.

	MRP+LRR group	MRP+LRL group	P-value
PTA (Pre-op and post-op change %)	70.4	76.2	0.009
PLS (Pre-op and post-op change %)	88.1	85.7	0.429
LPFA (Pre-op and post-op change %)	78.6	81.5	<0.001

When the effect of patella morphology on radiological results is evaluated, according to patella morphology, 13.33% (n=10) of knees were Type 1, 33.33% (n=25) of knees were Type 2, and 53.33% (n=40) of knees were Type 3. In the MRP+LRL group, 8.88% (n=4) of knees were Type 1, 35.55% (n=16) of knees were Type 2, and 55.55% (n=25) of knees were Type 3. In the MRP+LRR group, 20% (n=6) of knees were Type 1, 30% (n=9) of knees were Type 2, and 50% (n=15) of knees were Type 3. Both groups had no significant difference between patella type and pre-postoperative PLS, LPFA, CA, or PTA values (Table 2). There was no significant difference between the patella type and recurrence rate (p=0.343). Evaluating the effect of trochlea morphology on radiological results found no significant difference between pre-and postoperative change of the PTA (p=0.389), PLS (P=0.344), LPFA (P=0.489), CA (p=0.418), and trochlea type of both groups.

**Table 2:** Comparison of pre-operative and post-operative radiological measurements according to patella type. PLS: Patella lateral shift; LPFA: Lateral patellofemoral angle; CA: Congruence angle; PTA: Patellar tilt angle.

	Patella type
Pre-operative PLS (P-value)	0.678
Post-operative PLS (P-value)	0.875
Pre-operative LPFA (P-value)	0.665
Post-operative LPFA (P-value)	0.257
Pre-operative CA (P-value)	0.182
Post-operative CA (P-value)	0.515
Pre-operative PTA (P-value)	0.546
Post-operative PTA (P-value)	0.831

Clinical evaluation

In the MRP+LRR group, the mean Lysholm knee scale was 48.4 (range: 40-58) preoperatively and 74.2 (range: 56-90) postoperatively, and a significant increase was observed (p<0.001). The Kujala pain scale was found to be 46.17 (range: 40-55) preoperatively and 77 (range: 55-90) postoperatively, with a significant increase (p<0.001) observed. Tegner activity level scale was found to be 2.4 (range: 1-3) preoperatively and 5.6 (range: 3-8) postoperatively, with a significant increase (p<0.001) observed.

In the MRP+LRL group, the mean Lysholm knee scale was 50.82 (range: 36-58) preoperatively and 78.82 (range: 58-92) postoperatively, and a significant increase was observed (p<0.001). The Kujala pain scale was found to be 46.56 (range: 35-55) preoperatively and 80 (range: 55-90) postoperatively, with a significant increase (p<0.001) observed. Tegner activity level scale was found to be 2.64 (range: 1-3) preoperatively and 6.18 (range: 3-8) postoperatively, with a significant increase (p<0.001) observed.

A comparison of MRP+LRR and MRP+LRL groups found no significant difference between the pre-operative (p=0.205) and post-operative (p=0.228) Lysholm knee scales of both groups. There was no significant difference between the pre-operative (p=0.393) and post-operative (p=0.596) Kujala pain scales of both groups. There was no significant difference between the pre-operative (p=0.121) and post-operative (p=0.899) Tegner activity level scales of both groups (Table 3).

**Table 3:** Comparison of pre-operative and post-operative clinical evaluation scores. MRP+LRL: Medial retinaculum plication and lateral retinacular lengthening; MRP+LRR: Medial retinaculum plication and lateral retinacular release.

	MRP+LRL	MRP+LRR	P-value
Pre-operative Mean Lysholm knee scale	50.82	48.4	0.205
Post-operative Mean Lysholm knee scale	78.82	74.2	0.228
Pre-operative Mean Kujala pain scale	46.56	46.17	0.393
Post-operative Mean Kujala pain scale	80	77	0.596
Pre-operative Mean Tegner activity level	2.64	2.4	0.121
Post-operative Mean Tegner activity level	6.18	5.6	0.899

Complications

Revisions were performed on two knees in the MRP+LRR group and on two knees in the MRP+LRL group. There was no significant difference in the recurrence rates of both groups (p=0.157). A revision was performed on three knees because of the increase in PLS and PTA. MRP+LRR was applied to two of these three knees, and MRP+LRL was applied to one of them. Retrospective investigation of these patients found that the TT-TG distance was at the upper limit (18, 18, 19 mm). In revision, a tibial tuberosity anteromedialization osteotomy (Fulkerson osteotomy) was performed. In the case of the fourth patient, MRP+LRL was applied, and medial subluxation of the patella developed. Arthroscopic medial patellar release was performed in the revision. Hematoma developed in four knees with arthroscopic LRR and in one knee with LRL. No additional surgical procedure was required.

## Discussion

Pre-operative and post-operative PTA and LPFA changes were significantly better in knees with MRP+LRL compared to knees with MRP+LRR. There was no significant difference between the two groups in terms of PLS and CA changes before and after surgery. There was no significant correlation between patella and trochlea morphology and changes in PTA, LPFA, PLS and AA in both groups. While there was a significant difference between the pre-operative and post-operative changes in the Kujala pain scale, Lysholm instability scale, and Tegner activity scale in both groups, there was no significant difference between the two groups.

In patients with PF instability and without advanced osteochondral damage, treatment first provides neuromuscular control of the knee through conservative methods and physical therapy applications. It has been reported that surgical treatment yields satisfactory results in case persistent pain lasts for more than six months, despite conservative treatment lasting longer than six months [11]. The popularity of MRP, a proximal realignment surgery method, has declined in recent publications, though it is frequently used in current practice. It has been reported that methods that provide medial support to the patella increase quality of life when they are combined with lateral and distal realignment surgical methods [12]. Though indications for isolated use of LRR are very limited, its use has been accepted in the presence of patellar tilt and tight lateral retinaculum. However, it has often been applied in combination with other realignment surgery methods. Our study applied a combination of MRP and LRR.

A cadaver study in which isolated LRR was performed in PF instability reported that LRR was effective in centralizing the patella by regulating the extensor mechanism and relieving pain associated with lateral hypercompression syndrome [13]. A study on the application method of LRR found that with increasing flexion of the knee with PF instability, the patella begins to centralize, and the pressure on the lateral joint of the patella and trochlea increases. LRR aims to shift some of this load from lateral to medial. Paying attention to the reference points in the knee while performing LRR and not extending the LRR distance to the distal of the Gerdy tubercle and proximal to the VLO may prevent medial instability of the patella.

LRR has been used for many years to correct the extensor mechanism, and successful results have been reported. In the surgical technique, it is recommended not to extend the LRR to the proximal of VLO but to apply it from distal to proximal by checking the medial mobilization of the patella. However, it has been reported that LRL prevents medial subluxation of the patella better than LRR and is more successful in centralizing the patella to the trochlear sulcus and providing patellar tracking [14].

Many publications in the literature compare LRR and LRL. A study investigating the approach of experienced surgeons with a special interest in PF instability to LRR reported performing isolated LRR less often than isolated LRL. This was because isolated LRL was suggested to give better results than LRR in reducing lateral PF load and preventing chondral damage in patients with moderate patellar maltracking, lateral retinaculum, and capsule tension [15]. LRR and LRL are essential parts of the treatment in the correction of PTA in patella lateral compression syndrome and in PF instability, where lateral tension is a part of the pathology. It has been reported that LRL is safer than LRR as it preserves the lateral soft tissue integrity of the patella and prevents iatrogenic medial patellar subluxation [16]. Pagenstert G et al. reported that LRL gave better clinical results than LRR and caused less medial instability and quadriceps atrophy. This was also observed in the follow-up of knees with PF instability and lateral hypercompression syndrome due to lateral retinaculum tension and treated with LRR or LRL for more than two years [17]. Heyworth BE et al. performed revision with LRL in patients who had positive medial patellar glide test and had more than ¼ quadrant displacement of the patella to the medial side, despite the prior arthroscopic LRR application. After the revision, PF joint adjustment was achieved, the pain was significantly reduced, and functional scores were reported to improve significantly [18]. Many research results have reported findings on LRR and LRL in PF joint instability. Both methods have often been to complement combined surgical treatments. Insall JN et al. reported good and very good results with a rate of 91% in the 2-10 years follow-up of knees in which MRP and LRR were applied [19]. Scuderi GR reported good and very good results with a rate of 81% after MRP and LRR, and this result continued for up to nine years with a recurrence rate of 1.2% [20].

Satisfactory results have been reported regarding patellar re-luxation in knees with PF instability where open MRP and LRR are applied in combination therapy compared to arthroscopically applied MRP and LRR [21]. However, in a study applying MRP and LRR, re-dislocation and patient dissatisfaction were found at a rate of 20% in short-term follow-ups, and cartilage pathology was found at a rate of 43% in long-term follow-ups [22]. Despite the successful results of open and arthroscopic MRP techniques, few studies compare these techniques. In a study comparing arthroscopic MRP and open MRP techniques for PF instability in adolescents, the open technique yielded more successful results regarding the compatibility of PF joint alignment and functional results [23]. Our study applied open MRP because arthroscopic MRP is difficult to apply and has no accepted advantage other than cosmetic appearance.

MPFL reconstruction is the current topic of PF instability surgery. With the anatomical presentation of the medial patellar ligament structures more clearly, the tendency to reconstruct the ligaments instead of medial plication has increased [24]. However, this tendency has been exaggerated, and other major pathological factors in instability cases have not been adequately investigated in many publications [25]. Preferring the reconstruction of MPFL than its repair, the need for reconstruction with stronger ligaments than MPFL, and the problems caused by errors in the femoral attachment point have shown that there are details to be considered in this method [26].

Han H et al. reported adequate results in their study applying MPFL reconstruction and LRR [27]. However, a meta-analysis comparing knees with MPFL reconstruction and LRR and knees with MPFL reconstruction alone reported no difference in the results [28].

It has been reported that clinical results will be better when the adjustment angle in young male knees undergoing LRR is normalized [7]. Our study found LRR and LRL to give better results in young patients. It has been reported that the dramatic reduction in anterior knee pain, which is an important clinical finding in knees with PF instability, results from the denervation of the medial and lateral femoral cutaneous nerves in the surgical incision traces as well as the centralization of the patella in the femoral sulcus. For this reason, the pain of the same intensity was not reported in patients who developed patella redislocation after surgical treatment [29]. Therefore, in our study, four knees that developed patella re-dislocation and pain underwent revision.

Our study is different in its investigation of the relationship between patella and trochlea morphology and MRP+LRR and MRP+LRL. Furthermore, few studies exist in the literature comparing the radiological and clinical results of MRP+LRL and MRP+LRR.

The change in PTA and LPFA before and after surgery was significantly better in knees with MRP+LRL compared to knees with MRP+LRR. There was no significant difference between the two groups in terms of PLS and CA changes before and after surgery. We think that this difference may be due to the contraction that may develop during the healing of the lateral retinaculum defect with fibrous tissue after release. Both groups had no significant link between patella and trochlea morphology and changes in PTA, LPFA, PLS, and CA. A significant difference existed in the pre-and post-operative changes in the Kujala pain scale, Lysholm instability scale, and Tegner activity scale in both groups. However, there was no significant difference between the two groups.

There are some limitations in our study. Initially, the study was retrospective and based on medical records. The last control data of the patients were used. There is also the possibility that additional complications may have developed after these data. Secondly, radiological measurements were made by a single orthopedic specialist. It would be more reliable if it were evaluated by a second person and a radiologist.

## Conclusions

In conclusion, MRP+LRR or MRP+LRL provided successful results for correcting the instability in PF instability without osseous pathologies such as patella alta, TT-TG dysplasia, trochlea dysplasia, genu valgus, and tibial-femoral torsion. PTA, LPFA, and PLS values improved with both methods, but PTA and LPFA values improved more with the MRP-LRL method. Clinical results (Tegner activity level, Lysholm knee score, Kujala pain scale) were similar in both methods. There was no difference between the two methods in terms of post-operative complications and the need for revision surgery.
